# First reported quantitative microbiota in different livestock manures used as organic fertilizers in the Northeast of Thailand

**DOI:** 10.1038/s41598-020-80543-3

**Published:** 2021-01-08

**Authors:** Lampet Wongsaroj, Ratmanee Chanabun, Naruemon Tunsakul, Pinidphon Prombutara, Somsak Panha, Naraporn Somboonna

**Affiliations:** 1 Department of Microbiology, Faculty of Science, Chulalongkorn University, Phyathai Road, Pathumwan, Bangkok, 10330 Thailand; 2 Microbiome Research Unit for Probiotics in Food and Cosmetics, Chulalongkorn University, Bangkok, 10330 Thailand; 3 Program in Animal Science, Faculty of Agriculture Technology, Sakon Nakhon Rajabhat University, Sakon Nakhon, 47000 Thailand; 4 Program in Biotechnology, Faculty of Science, Chulalongkorn University, Bangkok, 10330 Thailand; 5 Omics Sciences and Bioinformatics Center, Faculty of Science, Chulalongkorn University, Bangkok, 10330 Thailand; 6 Department of Biology, Faculty of Science, Chulalongkorn University, Bangkok, 10330 Thailand; 7 Centre of Excellence on Biodiversity, Ministry of Higher of Education Science Research and Innovation/Faculty of Science, Chulalongkorn University, Bangkok, 10330 Thailand

**Keywords:** Microbiology, Biodiversity

## Abstract

Northeastern Thailand relies on agriculture as a major economic activity, and has used high levels of agrochemicals due to low facility, and salty sandy soil. To support soil recovery and sustainable agriculture, local farmers have used organic fertilizers from farmed animal feces. However, knowledge about these animal fecal manures remains minimal restricting their optimal use. Specifically, while bacteria are important for soil and plant growth, an abundance and a diversity of bacterial composition in these animal fecal manures have not been reported to allow selection and adjustment for a more effective organic fertilizer. This study thereby utilized metagenomics combined with 16S rRNA gene quantitative PCR (qPCR) and sequencing to analyze quantitative microbiota profiles in association with nutrients (N, P, K), organic matters, and the other physiochemical properties, of the commonly used earthworm manure and other manures from livestock animals (including breed and feeding diet variations) in the region. Unlike the other manures, the earthworm manure demonstrated more favorable nutrient profiles and physiochemical properties for forming fertile soil. Despite low total microbial biomass, the microbiota were enriched with maximal OTUs and Chao richness, and no plant pathogenic bacteria were found based on the VFDB database. The microbial metabolic potentials supported functions to promote crop growth, such as C, N and P cyclings, xenobiotic degradation, and synthesis of bioactive compounds. Pearson’s correlation analyses indicated that the quantitative microbiota of the earthworm manure were clustered in the same direction as N, and conductivity, salinity, and water content were essential to control the microbiota of animal manures.

## Introduction

Sustainable agriculture requires healthy soils, provided that soil microorganisms play significant functions in soil nutrient cycling, decomposition of toxic and complex organic molecules, and an increased crop fertility and health^1–5^. The northeastern region of Thailand is characterized by undulating terrains with four mountains (about 15% of the region’s total land surface) of a largely infertile, salty sandy soil type^6^. However, agriculture is the major economic activity in this region. Common cultivars include rice, cassava, rubber, sugarcane and corn. Subsequently, due to the inappropriate land area and soil type, the agriculture relies on agrochemicals. The Thai government’s Eleventh National Economic and Social Development Plan (2012–2016) reported that Thailand ranked first in the world for the use of registered chemicals in agriculture, without comment on the additional use of banned agrochemicals. Thai certified organic farmland accounted for only 0.3–0.5% of Thailand’s agriculture land^7^. Intensive use of agrochemicals include fertilizers that have been used to accelerate the production of plant products by providing mineral nutrients, including nitrogen (N), phosphorous (P) and potassium (K), and average 5–6 million tons were applied per year in Thailand during 2012–2016, of which 42% were used in rice farming^8^.

Chemical fertilizer, with defined inorganic formulations to match needs for different kinds of plant cultivars, cause imbalances in natural ecosystems. The application of chemical fertilizers, especially in poor soil with little ability to prevent leaching into groundwater, can cause adverse effects to the environment, such as waterway pollution, increased air pollution, and acidification and mineral depletion of the soil^8,9^. Long-term use also affects the soil microbial diversity and natural ecosystem^10–12^.

Organic fertilizers, including those from animal feces and plant waste composting, would seem preferable. Organic fertilizers from livestock feces contain decomposed complex molecules as nutrients and microbial diversity with the ability for decomposition. Hence, organic fertilizer provides, in addition to nutrients for plant growth and soil organisms, bacteria that may function to decompose and recycle agriculture wastes, and the long-term use was able to increase and modify soil microbial diversity. The manures (animal fecal composts) have contents and microorganisms to accommodate sustained arable soil bacterial communities^13^. Nevertheless, no knowledge of microbial and biophysiochemical compositions of the actual manures (type of species, breeds and diets) utilized by farmers in the northeastern region of Thailand is present. The assorted livestock species manures include *Perionyx excavatus* (earthworm, abbreviated as E), three breeds of *Gallus gallus* (Phuparn black-bone chickens) including black feather and bones breed (PC1), white feather but black skin and bones breed (PC2) and yellow feather but black skin and bones breed (PC3), three subspecies of *Bos taurus* (beef black cattles) including Phuparn cow (PCO), Charole cow (CC) and dairy cattle (DC), *Bubalus bubalis* (dairy Murrah buffalo, MB), *Capra aegagrus hircus* (goats) that were fed on (1) Pangola grass *Digitaria eriantha* (the highest quality of grasses for grazing with a high metabolizable energy, nutrients and protein level)^14^ (G1) or (2) Napier grass *Pennisetum purpureum* (the major livestock feed among ruminant animals in Thailand with a low nutrient digestibility and crude protein content)^15^ (G2), *Cervus timorensis* (Lucy deer, D), *Sus scrofa domesticus* (pig, S), and *Oryctolagus cuniculus* (rabbit, R). These animals have been predominantly farmed in the Northeast and their feces are used as organic fertilizers^16–18^. Yet, these livestock manures have given variable effects in promoting plant growth (Chanabun, personal communication).

The addition of manures to the soil has been reported to change the soil bacterial diversity, which had been affected from the long-term use of chemical fertilizers^12^. For example, Proteobacteria, Bacteroidetes, and Gemmatimonadetes became abundant in organic farm lands, and the presence of these bacteria were reported to correlate with an increased level of soil organic carbon (C) and nitrogen (N), and the total microbial biomass^17^. In contrast, Actinobacteria and Acidobacteria had become abundant in the agrochemical farm lands, where the soil was acidic and the chemically-grown crops more often had disease incidences^17–19^. Hence, organic fertilization is presumed to be one key to acquire a plant-beneficial bacteria community in the soil that is required for sustainable agriculture.

Consequently, understanding the diversity of indigenous microbial populations in diverse animal fecal manures that were commonly applied in the Northeast agriculture region of Thailand could provide insights into the possible impacts on the changes in soil microbial activities (affecting soil quality). This study, therefore utilized the advanced culture-independent approaches of 16S rRNA gene sequencing combined with quantitative PCR (qPCR) technologies to firstly identify and quantify the microbial compositions and diversities (quantitative microbiota), along with nutritional and physiochemical analyses, of the organic livestock fecal manures used in local farms in the Northeast of Thailand. The microbial metabolic potentials of each manure was also estimated from the quantitative microbiota. Together, the microbiome knowledge and the selection of appropriate animal fecal manure may help improve the soil quality management towards the sustained fertile lands.

## Materials and methods

### Livestock fecal manure collections

In total, 13 livestock fecal manure samples (E, PC1-3, PCO, CC, DC, MB, G1, G2, D, S and R), each with three independent random samplings at sites, as representative local organic fertilizers, were collected in the Phuparn Royal Development Study Centre area, Sakon Nakhon province, Thailand, in August 2018, between 11.00 and 15.00 h. This centre is a learning source for farmers to improve their own lands, in term of not only planning of agriculture (specifically rice, mushroom, para rubber, economic vegetables, mulberry, etc.) and livestock farming, but also water source and forest rehabilitation.

### Nitrogen (N), phosphorus (P), potassium (K), and fecal organic matter (FOM) compositions

The total N, P, and K contents in the fecal samples were measured following published protocols using a Rapitest Soil Test Kit 1601 (Luster Leaf Products, Inc., Illinois, USA)^20^. In brief, the fecal sample (5 g) was diluted 1:5 (w/v) in sterile water, mixed by vortexing for a minimum of 1 min, and allowed to settle overnight. The liquid suspension was harvested: 10 mL was used for the NPK measurements while 20 mL was used for the physiochemical properties. The 10 mL for NPK measurements was split into three aliquots, and the respective N, P or K powder (0.3 g) (Luster Leaf Products, Inc., Illinois, USA) was added to one aliquot (1 mL) and mixed by shaking for 30 s. The suspension was settled for 15 min, and the developed color was used to determine the N, P, or K amount (µg) based on comparison with the Rapitest Soil Test Kit 1601 manual’s color chart. In addition, the FOM content was measured in 1 g of each fecal sample using a Soil Organic Matter Test Kit (Kasetsart University Research and Development Institute, Bangkok, Thailand). Three independent measurements were performed to compute mean ± S.D.

### Physiochemical properties

The 20 mL fecal liquid suspension of each sample was used to analyse the pH, conductivity and salinity, using an Oakton PCD 650 Multiparameter with Calibration (GlobalTestSupply.com, North Carolina, USA). For measurement of water content in the sample, 2 g fecal sample was accurately weighted (wet weight; WW) and then dried in a microwave oven at 105 °C for 24 h^21^ to obtain the dry weight (DW). The percent water content was computed from 100 × (WW-DW)/DW. Three independent measurements were performed to compute mean ± S.D.

### Metagenomic extraction, and quantification of total bacteria copy number, *nirS *and *alkB*

Each sample (0.25 g) was extracted for metagenomic (microbial genomic) DNA using a DNeasy PowerSoil Kit, following the manufacturer's instructions (Qiagen, Maryland, USA). The quantity and quality of the extracted metagenomic DNA was analysed by 1% (w/v) agarose gel electrophoresis and nanodrop spectrophotometry (A260 and A260/A280, respectively). For a quantitative count of the total bacteria in copy unit, the 16S rRNA gene qPCR was performed using the universal primers 1392F (5′-GYACACACCGCCCGT-3′) and 1492R (5′-GGTTACCTTGTTACGACTT-3′)^22^. The qPCR thermocycling conditions were 95 °C 5 min, followed by 40 cycles of 95 °C 30 s, 55 °C 45 s and 72 °C 45 s, and ended with a melting curve analysis to validate a single proper amplicon peak (i.e. neither primer-dimer nor non-specific amplification)^23^. The reference for copy number computation was *Escherichia coli*, in which the 100 base pair (bp) 1392F-1492R amplicon fragments were cloned into pGEM-T-Easy Vector (Promega, Wisconsin, USA) and the recombinant plasmids were transformed into *E. coli* DH5α for expression. The inserted fragments were verified by colony PCR using the primers M13F (on vector) and 1492R (inserted fragment). Ten-fold serial dilutions of the extracted plasmids (10^4^–10^8^ copies/μL) were used as the references for the bacterial copy number computation based on Eq. (1)^23^:

1 $$ {\text{Copy number per}}\,\, \upmu {\text{L}} = \frac{{{\text{concentration }}({\text{ng}}/\upmu {\text{L}}) \times 6.023 \times 10^{23} \left( {\text{copies/mol}} \right)}}{{{\text{length (bp)}} \times 6.6 \times 10^{11} ({\text{ng/mol}})}}. $$

The reference DNA and metagenomic DNA (1 ng) were quantified by qPCR for bacterial copy number estimate using primers 1392F and 1492R, and iQ SYBR Green Supermix (Bio-Rad, California, USA) in a 20 µL total volume per well in a 48-well plate using the PCRmax Eco 48 real time PCR system (PCRmax, Staffordshire, UK). Three replicates were performed per reaction. For quantification of cytochrome-containing nitrate reductase (*nirS*) and alkane monooxygenase (*alkB*), established primers nirSF: 5′-GTSAACGTSAAGGARACSGG-3′ and nirSR: 5′-GASTTCGGRTGSGTCTTGA-3′, and alkBF: 5′-AACTACATCGAGCACTACGG-3′ and alkBR: 5′-TGAAGATGTGGTTGCTGTTCC-3′, along their thermal cycling parameters, were used^24,25^.

### 16S rRNA gene V3-V4 library preparation and MiSeq sequencing

PCR amplification of the 16S rRNA gene at the V3-V4 region was performed using the universal prokaryotic primers 515F (5′-GTGCCAGCMGCCGCGGTAA-3′) and 806R (5′-GGACTACHVGGGTWTCTAAT-3′) with appended adaptor and barcodes sequences as previously reported^26,27^. Briefly, each PCR reaction was comprised of 1 × EmeraldAmp GT PCR Master Mix (TaKaRa), 0.3 μM of each primer, and 50–100 ng of metagenomic DNA in a total volume of 50 μL. The PCR conditions were 94 °C 3 min, and 25 cycles of 94 °C 45 s, 50 °C 1 min and 72 °C 1 min 30 s, followed by 72 °C 10 min. A minimum of two independent PCR reactions were performed and pooled to prevent PCR stochastic bias. Then, the 381-bp amplicon was excised after agarose gel resolution and purified using a PureDireX PCR Clean-Up & Gel Extraction Kit (Bio-Helix, Keelung, Taiwan) prior to quantification using a Qubit 3.0 Fluorometer and Qubit dsDNA HS assay kit (Invitrogen, Waltham, USA). Finally, 180 ng of each barcoded amplicon product was pooled for sequencing using the Miseq300 platform (Illumina, San Diego, CA, USA), along with the sequencing primers and index sequence^26^. Sequencing was performed at the Omics Sciences and Bioinformatics Center, Chulalongkorn University (Bangkok, Thailand).

### Bioinformatic analyses for bacterial microbiota diversity and potential metabolisms

Raw sequences were processed according to Mothur version 1.39.1′s standard operating procedures for MiSeq^28^. Processes of screening for the quality sequences included removal of (1) short read lengths of ≤ 100 nucleotides (nt) excluding primer and barcode sequences, (2) ambiguous bases ≥ 8, (3) chimera sequences and (4) homopolymer of ≥ 8 nt. The quality sequences were aligned against the 16S rRNA gene databases SILVA version 132 to remove sequences of mitochondria, chloroplast, and eukaryotic lineages^29^, and Greengenes version 13.8 for prediction of taxonomy^30^. The sequences were classified into operational taxonomic unit (OTUs) based on the naïve Bayesian taxonomic method and default parameters (sequence similarity in OTU clustering was 78% for phylum, 88% order, 91% class, 93% family, 95% genus, and 97% species)^31^. Samples were normalized to an equal sequencing depth (7,945 quality sequences per sample), and the count of total bacteria from the 16S rRNA gene qPCR data were analyzed together with the percent microbiota composition from the 16S rRNA gene sequencing to yield the quantitative microbiota^32–34^.

Mothur version 1.39.1 was used to estimate the sequencing coverage (Good’s coverage index), rarefaction curve, alpha diversity (individual sample diversity: Chao richness and Shannon diversity), and beta diversity based on thetayc dissimilarity coefficients among the samples’ quantitative microbiota and two-dimension non-metric multidimensional scaling (NMDS)^28,35^. One-way ANOVA test was used to analyze the statistical significance for alpha diversity (p < 0.05), and Analysis of molecular variance (AMOVA) was used to analyze the statistical significance for beta diversity (p < 0.001). The frequencies of plant symbiosis bacteria and pathogenic bacteria were identified from the respective list of plant symbiosis (growth promoting) and plant pathogenic bacteria available in the Virulence Factor Database (VFDB)^36,37^, and compared among manure samples. Functional profiles of the microbial communities were predicted from the quantitative microbiota using PICRUSt (Phylogenetic Investigation of Communities by Reconstruction of Unobserved States), then the communities were clustered from the predicted functional profiles by unweighted pair group with arithmetic mean (UPGMA), and the functional profiles were statistically compared using STAMP (Statistical Analysis of Metagenomic Profiles)^38^. The metabolic functions were categorized by KEGG (Kyoto Encyclopedia of genes and genomes) pathways, level 2 representing categories of gene (COGs) and level 3 representing gene ontology (GO). Statistical tests of the differentially functional pathways between two communities were performed using a two-sided Welch’s test with Benjamini–Hochberg False Discovery Rate (FDR) multiple test correction method (p < 0.05).

## Results

### Nutrient, FOM, and physiochemical contents of the fecal manures

For the three main fertility nutrients (N, P and K) in the fecal manures (Table 1a) the E manure contained the highest N level at 80 µg/g, 16-fold more than the other samples. In addition, it had a high P level of 100 µg/g (along with PC1 and PC3) compared with an average of 34.66 µg/g for the others, and a moderate K level of 150 µg/g (others ranged from 125.00 to 350.00 µg/g. The FOM content ranged from 1.00 to 1.50% for all the animal manures (Table 1a). For the general physiochemical properties, chicken manures showed a relatively high salinity (Table 1b: 3.52 ± 0.28 to 4.01 ± 0.64 ppt), and these ionic salts caused a high conductivity (11.27 ± 0.69 to 12.79 ± 0.08 mS/cm) and high water saturation (73.29 ± 0.40 to 80.56 ± 3.17 mS/cm), giving less available water for plant roots. A moderate level of salinity, conductivity and water content, such as those in E, are generally preferred for farming soil. The water content of the manure was lowest in G2, goats fed with *P. purpureum* (23.75 ± 3.07%), but increased two-fold in G1 goats fed with *D. eriantha* (49.35 ± 6.08%), suggesting a different type of grass feed might confer different fecal liquid and dry contents, or the chemical composition influenced the water activity in the goat’s gut. The R manure also had a low water content (41.53 ± 5.57%).

**Table 1:** Characteristics of (a) NPK and FOM, and (b) physiochemical levels of the different animal fecal manures.

Fecal manures	Nutrient compositions (µg/g)^1^			FOM (%)
	N	P	K	
**(a)**				
PC1	< 5^2^	100.00 ± 0.00^d^	250.00 ± 50.00^b^	1.67 ± 0.29
PC2	< 5	40.00 ± 4.33^c^	208.33 ± 72.17^b^	1.37 ± 0.29
PC3	< 5	100.00 ± 0.00^d^	125.00 ± 0.00^a^	1.50 ± 0.00
PCO	< 5	35.00 ± 0.00^b^	125.00 ± 0.00^a^	1.17 ± 0.29
P	< 5	47.50 ± 4.33^c^	125.00 ± 0.00^a^	1.17 ± 0.29
R	< 5	41.67 ± 7.64^c^	333.33 ± 57.74^c^	1.17 ± 0.29
MB	< 5	33.33 ± 11.55^b^	300.00 ± 0.00^c^	1.00 ± 0.00
CC	< 5	28.33 ± 11.55^b^	266.67 ± 28.87^b^	1.37 ± 0.29
G1	< 5	48.33 ± 15.90^c^	300.00 ± 0.00^c^	1.37 ± 0.29
G2	5.00 ± 0.00	37.5 ± 3.53^b^	350.00 ± 70.71^d^	1.50 ± 0.00
DC	5.00 ± 0.00	20.00 ± 8.66^a^	283.33 ± 28.87^b^	1.00 ± 0.00
D	< 5	15.00 ± 0.00^a^	266.67 ± 57.74^b^	1.50 ± 0.00
E	80.00 ± 0.00	100.00 ± 0.00^d^	150.00 ± 43.30^a^	1.37 ± 0.29

Fecal manure	Salinity (ppt)^3^	Conductivity (mS/cm)	pH	Water content (%)
**(b)**				
PC1	4.01 ± 0.64^d^	12.79 ± 0.08^d^	8.16 ± 0.20 ^e^	73.29 ± 0.40^d^
PC2	3.52 ± 0.28^d^	11.27 ± 0.69^d^	7.43 ± 0.38^d^	80.56 ± 3.17 ^e^
PC3	3.95 ± 0.30^d^	11.99 ± 1.44^d^	6.94 ± 0.29^b^	80.43 ± 9.61 ^e^
PCO	1.70 ± 0.22^b^	5.40 ± 0.78^c^	6.78 ± 0.38^a^	78.05 ± 0.81^d^
S	1.83 ± 0.17^c^	6.20 ± 0.76^c^	7.22 ± 0.09^c^	71.66 ± 0.66^d^
R	1.04 ± 0.12^a^	3.28 ± 1.16^b^	6.37 ± 0.18^a^	41.53 ± 5.57^b^
MB	1.18 ± 0.11^b^	3.74 ± 0.32^b^	6.98 ± 0.20^b^	78.09 ± 0.61^d^
CC	0.88 ± 0.19^a^	3.06 ± 0.52^b^	7.11 ± 0.09^c^	79.00 ± 2.16^d^
G1	0.92 ± 0.28^a^	3.10 ± 0.85^b^	7.11 ± 0.28^c^	49.35 ± 6.08^b^
G2	0.61 ± 0.05^a^	1.72 ± 0.01^a^	7.22 ± 0.08^c^	23.75 ± 3.07^a^
DC	0.98 ± 0.31^a^	3.49 ± 0.47^b^	7.05 ± 0.13^b^	77.69 ± 2.38^d^
D	1.06 ± 0.12^a^	3.25 ± 0.36^b^	7.32 ± 0.05^d^	59.97 ± 1.60^c^
E	1.76 ± 0.13^c^	5.59 ± 0.39^c^	6.62 ± 0.10^a^	54.28 ± 1.17^c^

Different lowercase letter superscripts within the same column denote a significant difference (ANOVA, p < 0.05).

^1^Data are shown as the mean ± SD of three independent measurements.

^2^Measurement could not be specified when < 5 µg/g.

^3^ppt is parts per thousand.

The visual observation of each manure was in accord with the reported water content, with a dry and hard texture (insufficient moisture) for G1, G2, and R manures, and a wet texture for the chicken, cattle, pig, and buffalo manures. Too much water in the soil can adversely affect aeration, where the plant’s roots receive insufficient oxygen and rot. Moreover, most plants prefer a slightly acidic soil pH of 6.2–6.8, which was found in the R, MB and E composts (Table 1b). The manure pH can change through reactions such as organic matter decomposition that affects the availabilities of NPK^39^.

From the overall comparison of the NPK nutrients and physiochemical properties, the fecal manures from the Phuparn chicken species might be less suitable as a fertilizer because of the poor N level, and high level of salts and conductivity. Indeed, the manures from most species contained a low N level, and those from goat and rabbit also had a relatively low water content (insufficient water for a plant to absorb nutrients from the soil through the roots, to the trunk, leaves and fruits). A moderate water content in the manure was appropriate. But of course, a somewhat low water level could be adjusted by adding water. The biophysiochemical quality analyses of the manures suggested that the E manure would be appropriate as a fertilizer because of the enhanced NPK levels, low salinity, low conductivity, slightly acidic-neutral pH, and a moderate water content (Table 1b).

### Quantification of total bacteria in the fecal manures

**Figure 1:**
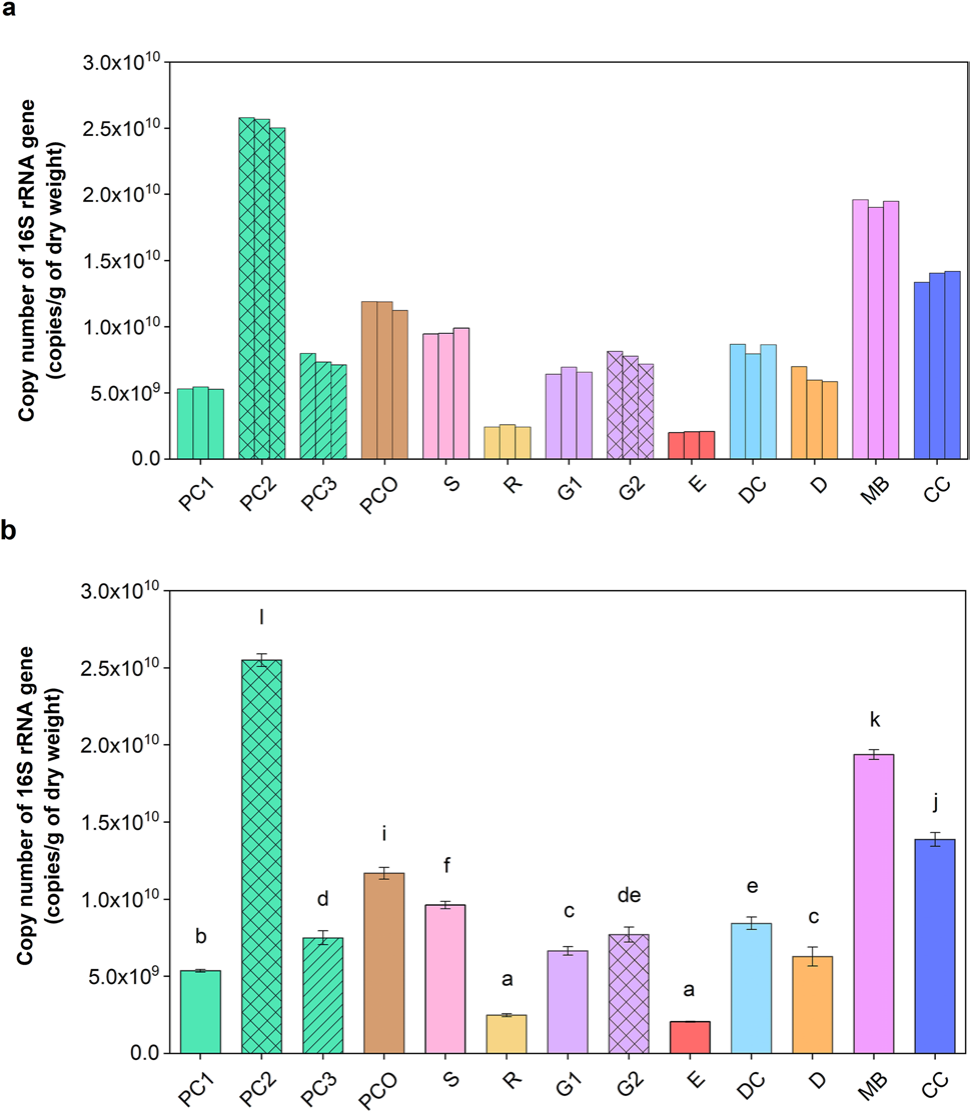
Quantification of 16S rRNA gene copies showing (**a**) the individual results from independent triplicate repeats and (**b**) mean ± S.D., in different livestock manure samples (per g DW) using qPCR. In (**b**), different letters above the bar indicated statistically significant differences among samples (one-way ANOVA with Waller Duncan test, p < 0.05).

The total number of bacteria in copies/g DW fecal manure was derived from the 16S rRNA gene qPCR. Figure 1a showed the data were consistent between independent triplicate samples, and that the E and R manures had the lowest bacterial counts (at 2.1 × 10^9^ and 2.5 × 10^9^ copies/g DW, respectively). The other animal fecal manures contained more than 5 × 10^9^ copies/g DW, and the greatest bacterial load was found in PC2 and MB at 2.6 × 10^10^ and 1.9 × 10^10^ copies/g DW, respectively, while PC1 and PC3 had a significantly lower level (p < 0.001 [PC1:PC2] and p = 0.001 [PC3:PC2]) (Fig. 1b).

### Bacterial taxonomic profiles by 16S rRNA gene sequences

The 16S rRNA gene V3-V4 library preparation and next generation sequencing were successful in that the number of raw and quality reads allowed data normalization (N = 7945 quality reads per sample) that covered > 99% and > 98% of the sequencing coverage of taxonomic compositions at the genus and species levels, respectively (Table 2). The average Good’s coverage indices were 99.54% and 99.43% for the genus and species levels, respectively (Supplemental Table 1). This was consistent with the plateau rarefaction curves, showing the frequencies of OTUs become constant despite increasing sequencing reads, meaning a sufficient sequencing coverage was obtained (Supplemental Fig. 1).

**Table 2:** Good's coverage indices (estimated sequencing coverage) and alpha diversity indices of bacterial taxonomic profiles by 16S rRNA gene sequences at the (a) genus and (b) species levels.

Fecal manure	Sample ID	Raw reads	Quality reads	OTUs	Good’s coverage	Chao	Shannon
**(a)**							
E	E_1	49,832	18,613	261	0.98930	380	3.16098
	E_2	33,624	12,371	309	0.98754	406.02	3.39099
	E_3	28,559	10,894	310	0.98628	437.95652	3.47094
PC1	PC1_1	301,641	196,013	148	0.99584	174.4	3.59382
	PC1_2	464,643	299,411	168	0.99484	198.37037	3.20792
	PC1_3	372,058	246,862	106	0.99648	129.625	2.38559
PC2	PC2_1	243,712	159,192	144	0.99509	218.1	2.66204
	PC2_2	270,242	176,288	155	0.99332	280.27272	3.02093
	PC2_3	276,023	178,737	127	0.99597	154.55555	2.76922
PC3	PC3_1	317,279	206,799	107	0.99672	132	3.19780
	PC3_2	371,787	237,351	116	0.99648	145.07692	3.27428
	PC3_3	275,511	183,585	107	0.99584	147.61538	2.07227
PCO	PCO_1	209,667	108,003	104	0.99698	134.66666	3.20418
	PCO_2	279,425	168,142	115	0.99622	140.58823	2.78577
	PCO_3	172,991	100,335	101	0.99761	109.55	2.89678
S	S_1	161,937	107,210	91	0.99622	163.5	2.80794
	S_2	320,353	205,017	83	0.99761	97.25	2.62696
	S_3	218,944	198,614	88	0.99648	135.25	2.66646
R	R_1	98,546	93,085	120	0.99610	153.21428	3.21580
	R_2	136,011	127,435	165	0.99383	234.17647	2.51931
	R_3	272,892	255,428	115	0.99622	146.07142	3.01602
G1	G1_1	127,942	112,607	99	0.99786	112.6	2.87956
	G1_2	44,671	16,998	84	0.99711	109.3	2.17222
	G1_3	82,404	29,752	92	0.99799	100	2.51211
G2	G2_1	24,100	8,688	93	0.99748	108.83333	2.38991
	G2_2	22,265	8,204	86	0.99786	105.42857	2.23782
	G2_3	20,917	7,945	100	0.99685	127.27272	2.57821
DC	DC_1	119,376	114,228	130	0.99560	165	2.37328
	DC_2	112,197	40,854	100	0.99685	142.85714	2.00946
	DC_3	148,943	137,401	105	0.99698	151.5	2.82303
D	D_1	154,153	97,954	136	0.99585	165.33333	3.21510
	D_2	201,247	117,168	141	0.99610	162.13636	3.25661
	D_3	211,853	115,864	150	0.99371	222.05882	3.29154
MB	MB_1	146,008	135,114	126	0.99484	189.07692	2.82331
	MB_2	134,925	128,005	146	0.99585	173.78947	3.04006
	MB_3	132,775	125,347	124	0.99622	157.46153	2.68982
CC	CC_1	186,002	173,315	182	0.99396	225.38461	3.39068
	CC_2	130,352	122,980	121	0.99597	148.55555	2.07403
	CC_3	180,039	171,258	119	0.99610	161.27272	2.81354

Fecal manure	Sample ID	OTUs	Good’s coverage	Chao	Shannon		
**(b)**							
E	E_1	293	0.98754	396.2128	3.21184		
	E_2	332	0.98351	521.2222	3.38902		
	E_3	325	0.98540	496.0256	3.46138		
PC1	PC1_1	175	0.99358	230.4348	3.66648		
	PC1_2	185	0.99446	230.0476	3.34555		
	PC1_3	123	0.99534	174.2308	2.44652		
PC2	PC2_1	162	0.99421	211.2857	2.68645		
	PC2_2	167	0.99308	249.5	3.02509		
	PC2_3	145	0.99421	214	2.92657		
PC3	PC3_1	131	0.99459	195.5	3.31345		
	PC3_2	130	0.99622	163.4615	3.39241		
	PC3_3	114	0.99522	177.9091	2.26757		
PCO	PCO_1	140	0.99408	200.0556	3.37754		
	PCO_2	139	0.99471	200.5	2.94793		
	PCO_3	122	0.99534	182.5455	3.09510		
S	S_1	115	0.99522	193.1111	2.84378		
	S_2	109	0.99585	157	2.77461		
	S_3	98	0.99610	140.2727	2.74297		
R	R_1	132	0.99610	157.8333	3.30997		
	R_2	189	0.99295	248.2308	2.78897		
	R_3	127	0.99597	165.1538	3.10773		
G1	G1_1	130	0.99559	172.5	2.93511		
	G1_2	92	0.99698	126.5	2.22541		
	G1_3	112	0.99648	146.3636	2.523160		
G2	G2_1	106	0.99660	129.4	2.40532		
	G2_2	96	0.99736	115.0909	2.25199		
	G2_3	119	0.99509	180.75	2.59913		
DC	DC_1	134	0.99534	185.2308	2.49933		
	DC_2	118	0.99547	163	2.06601		
	DC_3	109	0.99723	120	2.82375		
D	D_1	165	0.99396	210.12	3.28698		
	D_2	178	0.99245	266.5	3.32280		
	D_3	166	0.99320	237.55	3.34855		
MB	MB_1	126	0.99660	140.04	2.89128		
	MB_2	176	0.99358	217.129	3.06995		
	MB_3	161	0.99383	217	2.82487		
CC	CC_1	199	0.99333	258.913	3.49072		
	CC_2	131	0.99547	166	2.33934		
	CC_3	142	0.99534	177.0526	2.91837		

**Figure 2:**
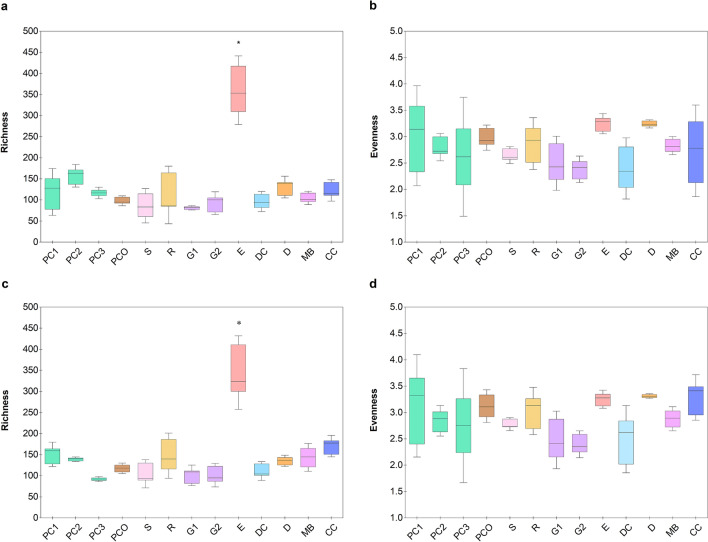
Alpha diversity measurements of OTU compositions at (**a**,**b**) genus and (**c**,**d**) species levels, by richness (Chao) and evenness (Shannon). Box plot with bar representing the mean from three sequencing replicates, and asterisk (*) indicates a statistically significant difference by one-way ANOVA at p < 0.05.

Combining the count of total 16S rRNA gene copies with the OTU percent compositions gave the quantitative number of copies of each OTU in a community. These quantitative microbiota data were then used to compute the alpha and beta diversity measurements. The alpha diversity revealed that the microbiota of E had the relatively most diverse OTUs at both the genus and species levels (Chao richness, Fig. 2a,c), regardless of having the lowest total bacterial count (Fig. 1). The low total bacterial abundance but high diversity in E underlined that the various OTUs of bacteria in E might be present in small numbers when compared to the copy numbers of OTUs in the other animal manures. Note that the alpha diversity obtained by considering the distribution of general OTUs among the different animal manures were similar (Shannon diversity, Fig. 2b,d).

**Figure 3:**
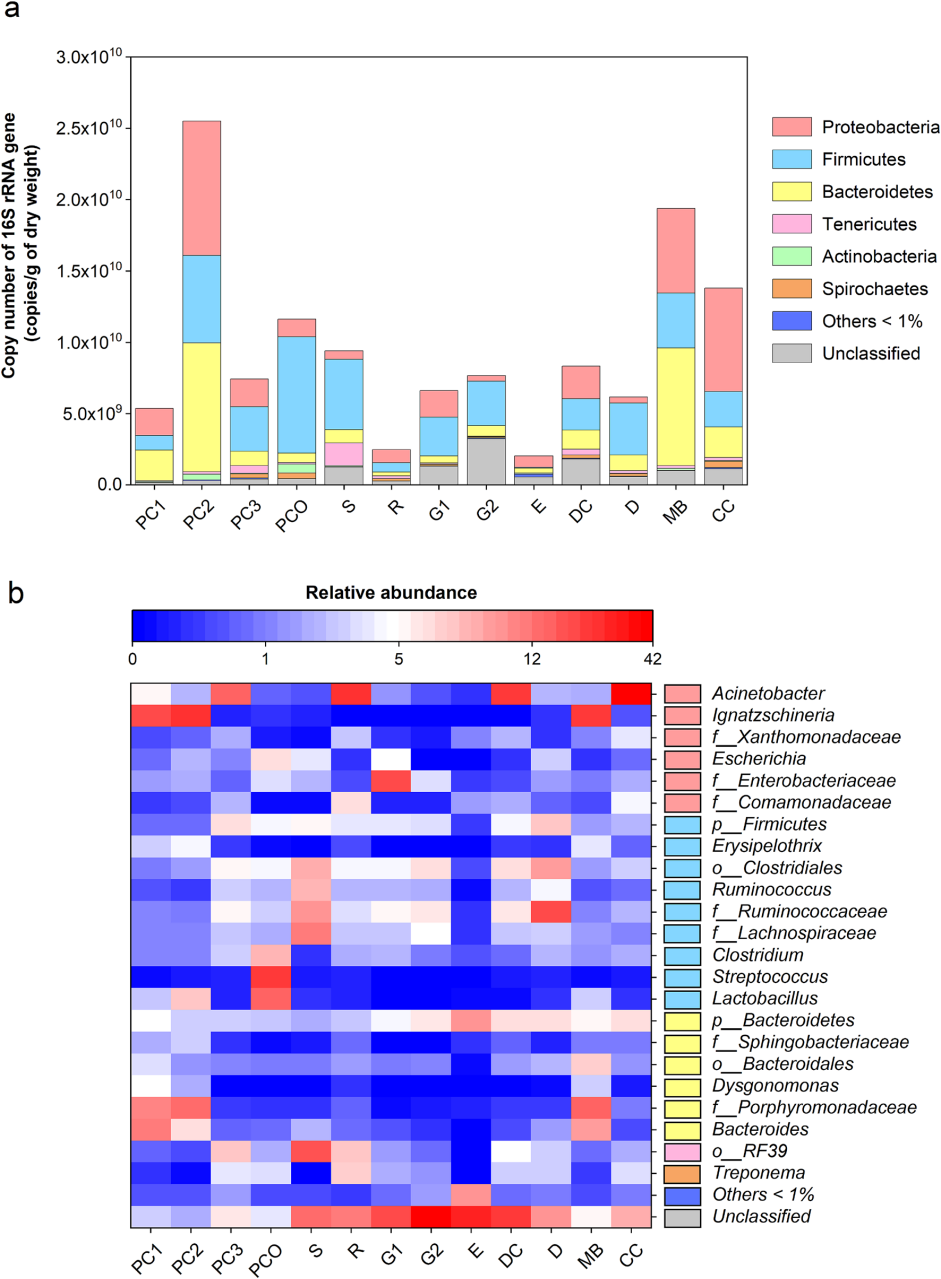
Analyses of bacterial community structures by (**a**) copy number of each phylum and (**b**) percent abundance of each genus (heatmap showed only genera with > 1% abundance). In (**b**), the OTUs where Mothur could not identify the genus name were denoted by small letters (*p_* abbreviates phylum; *o_*, order; and *f_*, family) to the deepest taxonomic names that could be identified.

Figure 3a described the quantitative number of bacterial phylum OTUs. Proteobacteria, Firmicutes and Bacteroidetes were common in all manure samples. PC2 and CC were predominantly Proteobacteria (at 9.41 × 10^9^ and 7.24 × 10^9^ copies/g DW, respectively), and MB and also PC2 were predominantly Bacteroidetes (at 8.22 × 10^9^ and 9.03 × 10^9^ copies/g DW, respectively). Firmicutes were also moderately common in all the manures except for E. The percentage abundance of genera above 1% were demonstrated in Fig. 3b, and was comprised of a total of 23 genera. The genus *Ignatzschineria* were responsible for the high Proteobacteria levels in PC2, MB and PC1, while the genus *Acinetobacter* were responsible for the high Proteobacteria levels in the other animal manures except for the E. Manures PC1, PC2 and MB contained all Bacteroides genera. *Streptococcus* was only found in PCO and was relatively abundant (20.04%). *Escherichia* were relatively low in R, G2, E, DC and MB, while *Treponema* were responsible for the high Spirochaetes levels in PC3, PCO and R at 3.63, 3.20 and 6.75%, respectively. Noted that the diversified OTUs in E belonged to Bacteroidetes at 6.28%. Moreover, a proportionate percentage of unclassified OTUs were presented in G1, G2, DC and E at 19.62, 41.86, 21.71 and 27.22%, respectively.

### Relative frequencies of plant symbiotic and pathogenic bacterial genera

**Figure 4:**
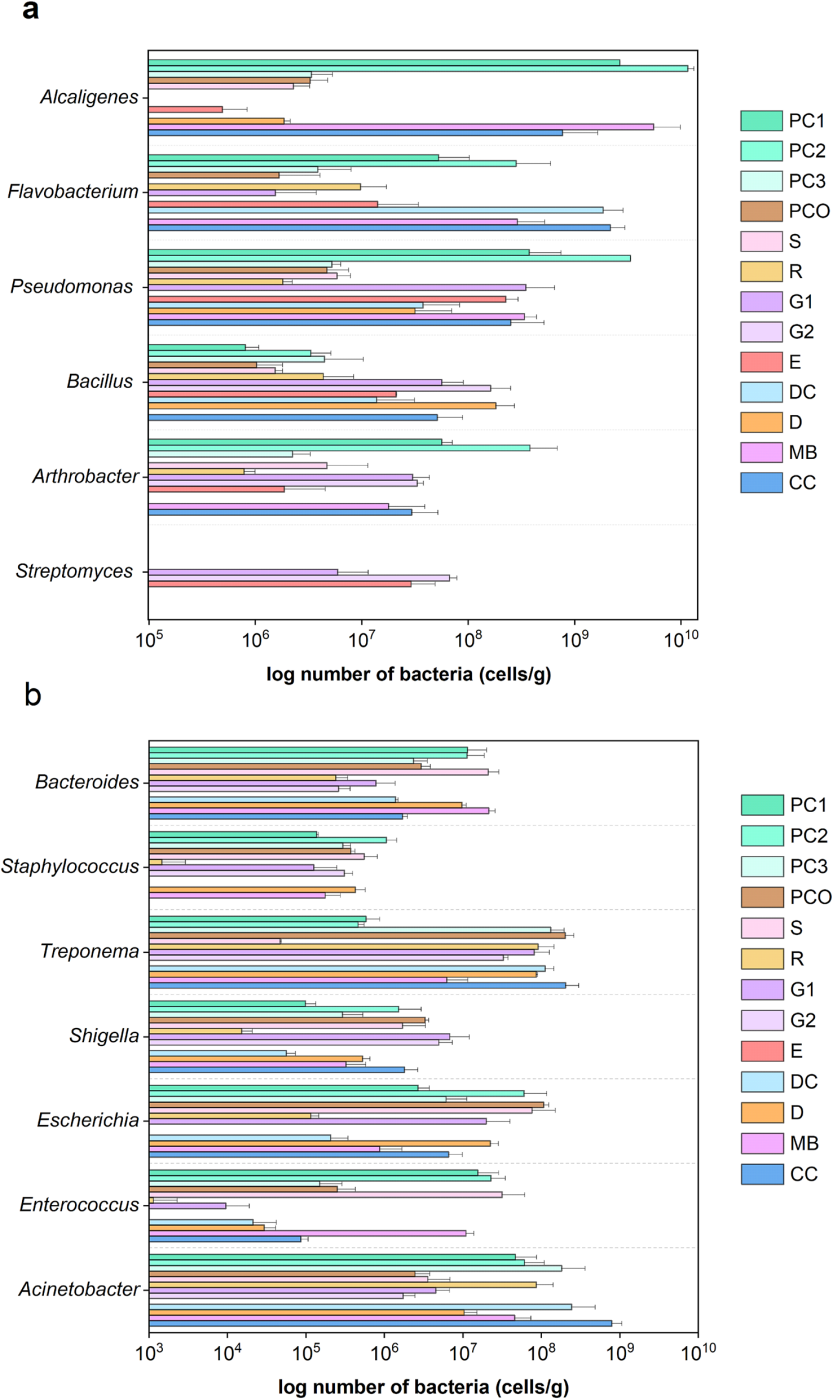
Comparative number of bacterial genera categorized as plant (**a**) symbiotic or (**b**) pathogenic genera across the different animal manures. Data represented mean ± S.D. List of bacteria categorized as plant symbionts and pathogens were downloaded from the Virulence Factor Database (VFDB).

Plant symbiosis and pathogenic bacteria were analysed across the different animal manures. Manures PC1-3, G1, G2 and CC demonstrated generally abundant symbiotic bacteria comprised of the genera *Pseudomonas*, *Bacillus*, *Arthrobacter*, *Flavobacterium*, *Alcaligenes* and *Streptomyces* (Fig. 4a). For examples, PC2 contained abundant *Alcaligenes* (1.16 × 10^10^ cells/g DW), *Pseudomonas* (3.35 × 10^9^), *Flavobacterium* (2.82 × 10^8^) and *Arthrobacter* (3.8 × 10^8^). *Streptomyces* was only found in G1, G2 and E in moderate numbers (5.9–29.0 × 10^6^ cells/g DW).

For the plant pathogenic bacteria, high levels were found in the manures that also contained high levels of plant symbiosis bacteria (PC1-3, G1, G2 and CC), except for E, plus the other manures, such as S, D and MB. The E had none of VFDB-listed plant pathogens. Thus, all manures except for E contained plant pathogenic bacteria, and these were from *Escherichia*, *Shigella*, *Enterococcus*, *Clostridium*, *Acinetobacter*, *Treponema*, *Staphylococcus* and *Bacteroides* (Fig. 4b). This finding correlated with the percent abundance of genera (Fig. 3b) where, examples, *Treponema* were relatively high in PCO and PC3 at 2.03 × 10^8^ and 1.31 × 10^8^ cells/g DW, respectively. *Escherichia* were relatively low in R and DC at 1.15 × 10^5^ and 2.06 × 10^5^ cells/g DW, respectively. In contrast, only E did not contain any VFDB-listed plant pathogenic bacteria suggesting that E offers a plant pathogen-free organic fertilizer.

### Relationship among bacterial communities, and statistical correlation with the biophysiochemical properties

The NMDS demonstrated both the reproducibility of the data between the independent triplicate samples, except for R and PCO. The larger variation in bacterial communities were found generally between the manures from different animal species, while the minor variation in bacterial communities were found between the breeds, or the feeding diets. For examples, the variation in quantitative microbiota profiles between G1, G2 and E, compared with PC1-3 (p = 0.085) and MB (p = 0.59) (Supplemental Fig. 2). Indeed, the quantitative microbiota structures belonging to the six animal manures that had all the VFDB’s categorized plant pathogens (PC, S, R, D and MB, except PCO) were rather distant from E (p = 0.101, 0.052, 0.089, 0.104 and 0.099, respectively).

**Figure 5:**
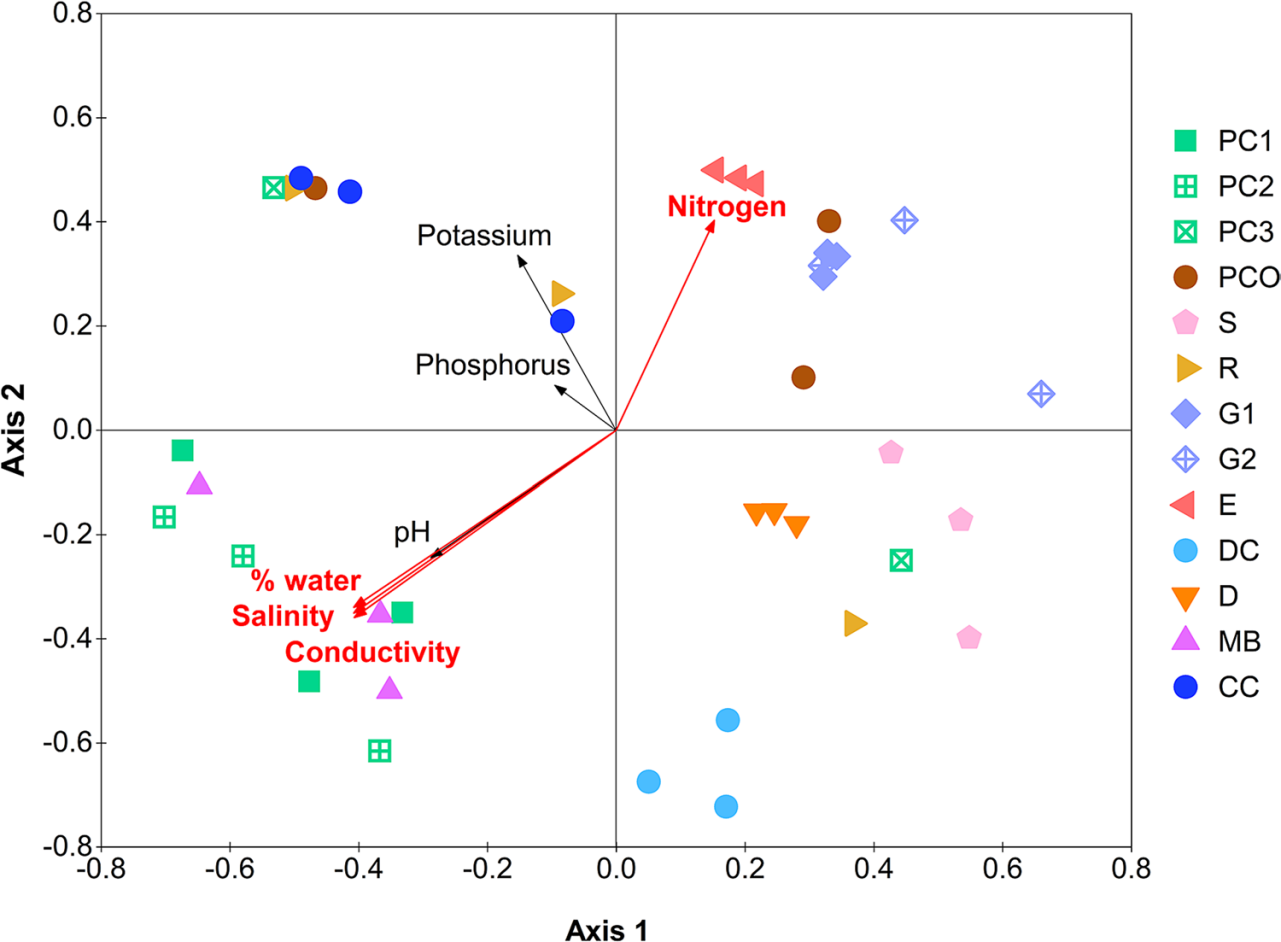
Relationship among bacterial communities via NMDS constructed from thetayc distance coefficients among quantitative microbiota (stress value = 0.15, R^2^ = 0.86), and Pearson's correlation with nutrients and physiochemical properties (AMOVA, p < 0.001). A vector direction and length represented the direction and strength of that nutrient or physiochemical factor to the communities. A red arrow with red font indicated a statistically significant correlation (p < 0.05).

Seven parameters (NPK and four physiochemical properties) were analyzed for possible Pearson’s correlation with any of the quantitative microbiota structures. The N level was strongly correlated (p = 0.01) to the structures and in the same direction of the E (Fig. 5). The percent water, salinity and conductivity characteristics were significant and associated in the direction opposite to E, and also to the G1, G2 and PCO microbiota structures. On the other hand, the PC1, PC2 and MB microbiota structures were strongly associated with the water content, salinity and conductivity. These parameters are suggested to be important in controlling the diversity of microbiota structures.

### Predicted functional profiles from quantitative microbiota

**Figure 6:**
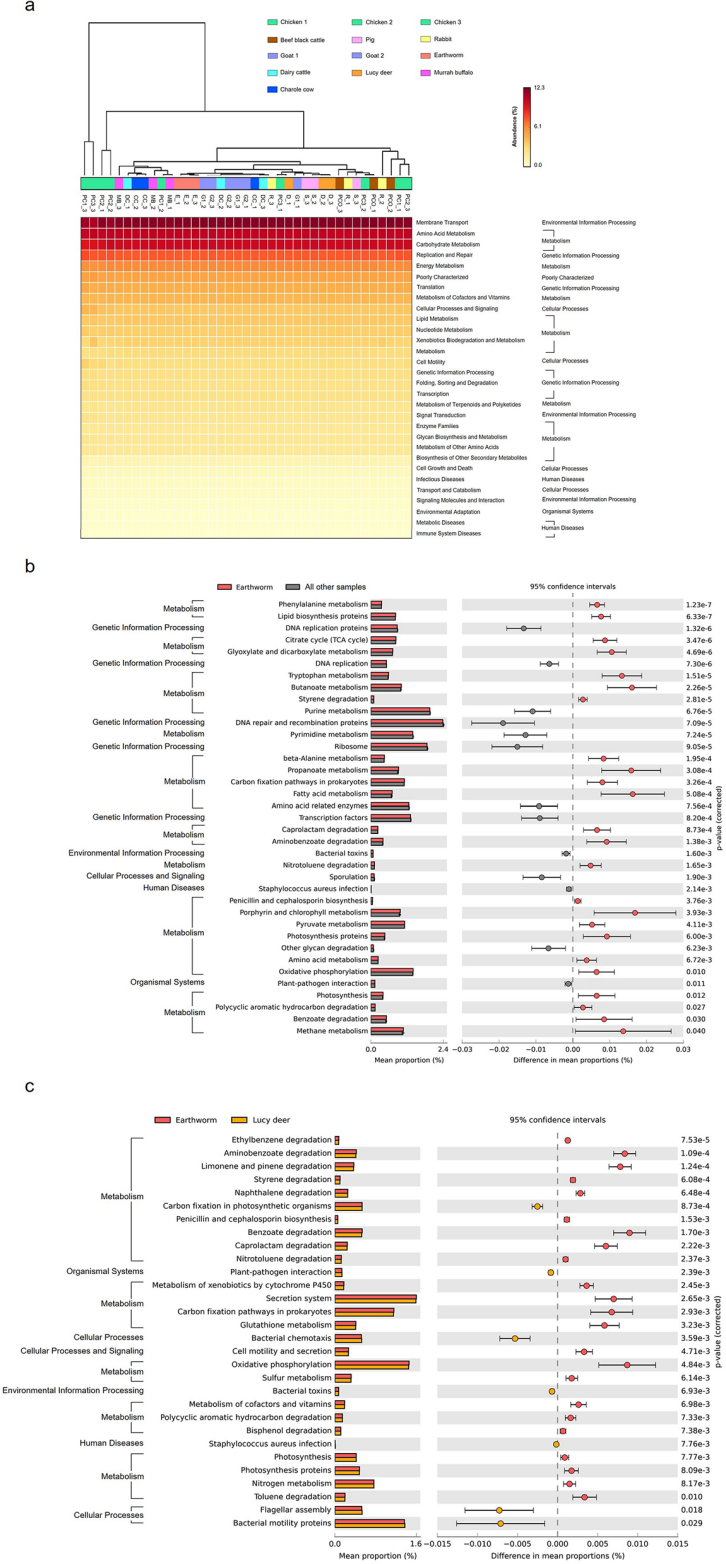
Predictions of functional profiles into KEGG levels 2 and 3 from quantitative microbiota using PICRUSt and STAMP. (**a**) On the top showing the clustering of functional profiles of manure samples by UPGMA. The significantly different KEGG functions between (**b**) the earthworm and the average from the other samples, and (**c**) the earthworm and Lucy deer, were compared using two-sided Welch's *t*-test (corrected p < 0.05).

Clustering by metabolic profiles separated most of the PC communities, then PCO and CC, from the E clusters (Fig. 6a). The quantitative microbiota in E demonstrated enhanced levels of COGs defined as metabolism, for instances, carbon fixation, oxidative phosphorylation and photosynthesis pathways compared to the rests (Fig. 6b: p = 3.26 × 10^−3^, 0.01 and 0.012, in order). The degradation functions of toxic compounds (xenobiotics) including styrene, caprolactam, aminobenzoate, nitrotoluene, polycyclic aromatic hydrocarbon and benzoate were also high in E compared to the rest (p = 2.81 × 10^−5^, 8.73 × 10^−4^, 1.38 × 10^−3^, 1.65 × 10^−3^, 0.027 and 0.03, respectively), indicating that the earthworm manure had a greater potential potency to degrade toxic compounds contaminated in the soil.

The KEGG categories of bacterial toxin, *Staphylococcus aureus* infection, and plant-pathogen interaction were dominated statistically higher in the other manures than E (p = 1.6 × 10^−3^, 2.14 × 10^−3^ and 0.011, respectively), in which the findings were correlated with Fig. 4b. Within the same metabolic profile clusters with E (Fig. 6a) but carried all VFDB listed plant pathogens (Fig. 4b), the metabolic profile of D was selected for comparison with that of E to perhaps capture some metabolic differences that might involve pathogens. Consistently, anti-bacterial compounds (i.e. biosynthesis of penicillin and cephalosporin, and antibiotic secondary metabolites) were higher in E (Fig. 6c), supporting the ability of the manure’s bacterial community to potentially protect against plant pathogens40. Related plant pathogen functions (bacterial toxin, *Staphylococcus aureus* infection, and plant-pathogen interactions) were statistically higher in D (Fig. 6c), resembling those in the other manures (Fig. 6b: all other samples). Moreover, E had enhanced levels for carbon fixation and photosynthesis, which are essential for plant growth (p = 2.93 × 10^−3^ and 7.77 × 10^−3^, respectively).

## Discussion

Organic manures have been valued as one approach to improve soil quality for planting among Thai local farmers. This study contributes a novel understanding of the assorted range of different local livestock manures by their quantitative microbiota compositions using the advanced next generation sequencing, associated with the manures’ nutrients and biophysiochemical properties. The NPK measurements revealed that the E manure had high organic N and P levels, consistent with previous reports that earthworms in soil increased N, P and C nutrients to support soil quality for plant growth^41,42^. The elevation of N and P levels in soil could also shift the soil bacterial communities in a way to support common and copiotrophic bacteria (e.g. *Actinobacteria* and *Alphaproteobacteria*), while limiting *Acidobacteria* and *Planctomycetes*^19,43^. This is consistently related to our study where, for example, the E manure showed relatively higher *Actinobacteria* and *Alphaproteobacteria* levels (4.89 × 10^9^ and 2.55 × 10^9^ cells/g DW, respectively) than those in the manures of the other animals. Although, the NPK and FOM contents in E might be lower than agrochemical fertilizers, the organic minerals in E are safe for use in the soil and our environmental ecosystems, and can add bacterial diversity to the chemically damaged soil.

Selection of the appropriate physiochemical properties (pH, salt, and ions) is essential for soil fertility. The high salinity and electrical conductivity in the PC1-3 manures compared to the others, suggested that chicken feces might not be an appropriate manure because the high ionic salts could interfere with the plant’s water absorption and also cause an imbalance in nutrient ions. A dramatically low soil pH (< 5.5) was caused from the long-term chemical fertilizer treatment. Here, none of the tested animal feces (manure) exhibited an acidic pH. Rather, they exhibited a near neutral range pH of 6.2–6.8 in E, R and MB which was optimal for a fertile soil, given the slight decrease of pH in E with its high N nutrient content could be due to an incomplete N cycling activity leading to acidic derivative^44^.

The lower total bacterial biomass, yet higher alpha diversity (Chao, OTUs’ richness index) in E compared to the other manures supported the fact that the earthworm gut lacks sufficient enzymes to digest OMs, but rather the earthworm gut selects and stimulates microorganisms (thus the reduced total microbial biomass in the E) to assist in the digestion of OMs. These include denitrifying bacteria that are able to survive in the reduced oxygen condition of the earthworm gut^41,45,46^. Further, the relatively high level of available N and P nutrients in the E manure would increase the species richness by promoting copiotrophic microorganisms^41^.

Overall, Proteobacteria, Firmicutes and Bacteroidetes were found to be common phyla in all the manures (avg. 16.7, 31.3 and 15.9%, respectively), which was consistent with previous reports. For instances, the fecal microbiota of horse, minipig and conventional pig were mainly comprised of the phyla Firmicutes and Bacteroidetes^23,47^. The low (3.3%) content of Firmicutes, gram-positive bacteria that can survive in desiccated and extreme conditions, found in E supported an amiable fecal compost along with the NPK availabilities, low salinity, and conductivity; hence the low presence of Firmicutes were present. The presence of Proteobacteria might be due to available nutrients (NPK and C) in animal feces to support their growth^19^, where Proteobacteria promote nutrient cycling to support soil bacterial growth.

In addition, the microbiota in E were found to be plant pathogen-free, and were comprised of every genus listed as plant growth promoting bacteria, for examples, *Pseudomonas*, *Streptomyces* and *Flavobacterium*. These bacteria function to assist soil nutrient cycling and plant growth, such as *P. fluorescens* and *P. putida* process nitrogen fixation and phosphate solubilization, and *Flavobacterium* processes (solubilizes) complex organic substrates to simple forms for other species usages^37,48^. Subsequently, the presence of plant growth-promoting bacteria was linked to the ability to support crop growth. Manures PC1-3 also contained high numbers of plant-growth promoting bacteria (except for *Streptomyces*), but had a number of plant pathogens similar to the other animal manures (except for E). Plant-pathogenic bacteria, such as *Escherichia* (e.g. *E. coli*), *Shigella* and *Acinetobacter*, come from soil and animal fecal contaminations. Many species of *Escherichia* and *Shigella* synthesize toxins, and some have been reported to be antibiotic-resistant (e.g. *Acinetobacter baumannii*), reducing the success of plant treatment by antibiotics^49^. *Treponema* cause chronic venereal disease (syphilis). Thus, even the minor abundance of some plant pathogens in fecal composts might be hazardous as an organic manure to enrich soil fertility for crop growth. Consistently, previous studies reported that E enhance plant growth and soil biodiversity by promoting beneficial bacteria, which could directly benefit plants via production of plant growth-regulating hormones and enzymes, or indirectly via secondary metabolites that control plant pathogens, nematodes and other pests^40^. However, our study analysis was limited to bacteria, while plant pathogens may be fungi.

In support of E, we observed rice and lemon planting results in our sampling local farms, and found that rice kernels supplemented E were relatively healthy with fewer leaves, while those supplemented with CC, for example, were withering with many leaves. Furthermore, many leaves could pose a risk for one of the most rice pest named brown planthopper that directly damages rice cultivars by feeding and transmitting two viruses, ragged stunt virus and rice grassy stunt virus. For lemon cultivars, using E was found relatively high in flower stalking and fruiting (Chanabun, personal communication). Studies reported that earthworms’ guts (including gut microbial communities) can digest various types of organic and even agro-industrial wastes into the reduced organic carbon forms, which represent the more ready-to-use N, P and K forms for plants and soil microbiota^50^. The vermicompost was thereby considered a powerful biofertilizer in sustainable agriculture, containing not only nutrients but also potentially beneficial microbial communities^41^. Indeed, the presence of earthworms represents one biological indicator of naturally arable soil, because its living could accelerate a soil bioconversion process by 2–5 times faster compared to traditional composting (Chanabun, personal communication). Subsequently, we analyzed two key microbial functional gene expressions in E that involve a soil bioconversion process, a cytochrome-containing nitrite reductase (*nirS*) and an alkane monooxygenase (*alkB*). The *nirS* functions in denitrification in nitrogen cycling and *alkB* functions in alkane biodegradation, respectively. Comparing among E, D and MB, E had the relatively greatest *nirS* and *alkB* copies while the total bacterial copy number (*16S rRNA*) was lowest (Supplemental Fig. 3). This finding was consistent with the high metabolisms of “nitrogen metabolism” and “alkene degradation” predicted from the E’s quantitative microbiota using PICRUSt (Fig. 6b,c). The PICRUSt algorithm has been used to predict the functional features of the bacterial community in environmental samples, including soil and fecal manure. For instances, Wang et al*.*^51^ analyzed metabolism functions of bacterial community from microbiota data using PICRUSt in different phases of a swine composting system. In 2019, Meng et al*.* utilized PICRUSt to analyze metabolic potentials from daily livestock manure microbiota and found that metabolisms of amino acids, lipids and carbohydrates were similar to the previous reports^52^. Therefore, our E quantitative microbiota and functional analyses revealed the understanding of the E.

Different earthworm species have previously been reported to contain different bacterial richness and diversity^41^. This study reported the influences that affect the microbiota diversity and microbial metabolic potentials in livestock manures that were found different between animal genera and species (e.g. PCO vs. CC), and animal feeds (G1 vs. G2). Analyses of statistical correlations suggested the importance of available N, conductivity, salinity, and water content, in regulating the quantitative microbiota structures and the microbial metabolic functions. Worthy for our further ongoing studies are effects of (1) these organic manure farmlands compared with long-term chemical farmlands, (2) different species of earthworms, and (3) the appropriate feed diets and beddings for earthworm manures (fecal and urea composts).

## Conclusion

Total microbial biomass and microbial community structures were characterized from the manures of different livestock species and breeds, and livestock diets. These fecal manures showed differences in available nutrients and physiochemical properties that could affect the soil fertility for crop growth. The most appropriate livestock manures among the different animal species and feeds analyzed in this study was the E manure due to its appropriate fecal composition, as well as the bacterial communities, which consisted of several plant promoters but no plant pathogens, and metabolic potentials that involved many nutrient cyclings, complex phosphate or other xenobiotics degradation, and bioactive compounds. The favorable microbial community in this E manure is expected to support soil fertility and crop health.

## Supplementary Information

Supplementary Information

## Data Availability

Nucleic acid sequences in this study were deposited in an NCBI open access Sequence Read Archive database, accession number SRP246309.
