# Supplementary material for: First reported quantitative microbiota in different livestock manures used as organic fertilizers in the Northeast of Thailand
Source: Sci Rep. 2021 Jan 8;11:102. doi: 10.1038/s41598-020-80543-3 (PMC7794567; doi:10.1038/s41598-020-80543-3)
Supplement: Supplementary file 1 — Supplementary Information [file 41598_2020_80543_MOESM1_ESM.docx]

**First reported quantitative microbiota in different livestock manures used as organic fertilizers in the Northeast of Thailand**

Lampet Wongsaroj^a,b^, Ratmanee Chanabun^c^, Naruemon Tunsakul^d^, Pinidphon Prombutara^b,e^, Somsak Panha^f,g^, Naraporn Somboonna^a,b^*

**Supplementary Figures and Tables**

**Supplemental Figure 1.** Rarefaction curves displaying the species richness (Y axis, OTUs) at various taxonomic levels, from phylum to species, which reached the plateau at the normalized sequencing depth.


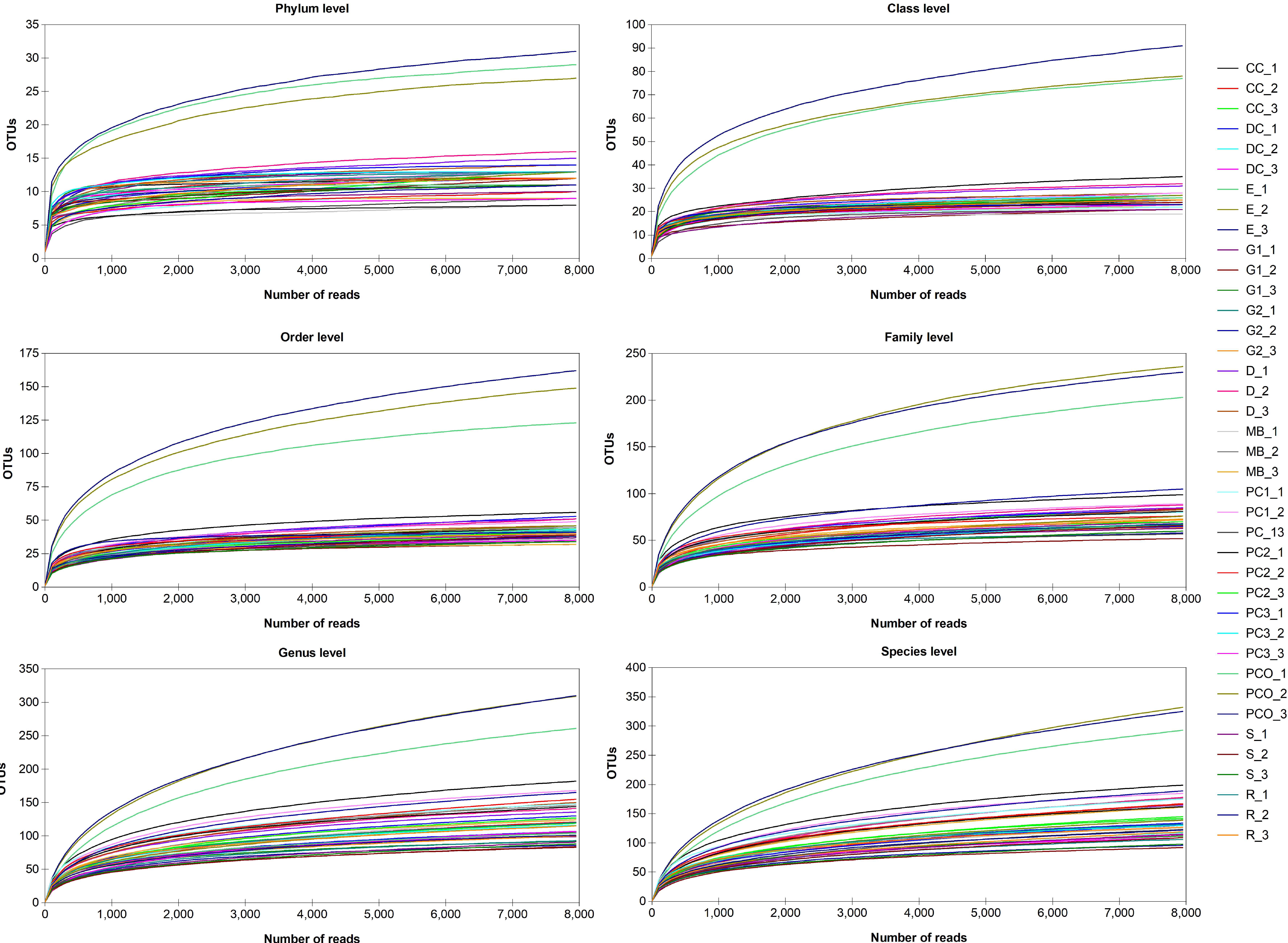


**Supplemental Figure 2.** NMDS constructed from thetayc distance coefficients among quantitative microbiota, provided that manures that contained no pathogenic bacteria (E manures) were circled blue, and those that contained all the pathogenic bacterial genera listed in VFDB were circled brown (PC1-3, MB, S and D).


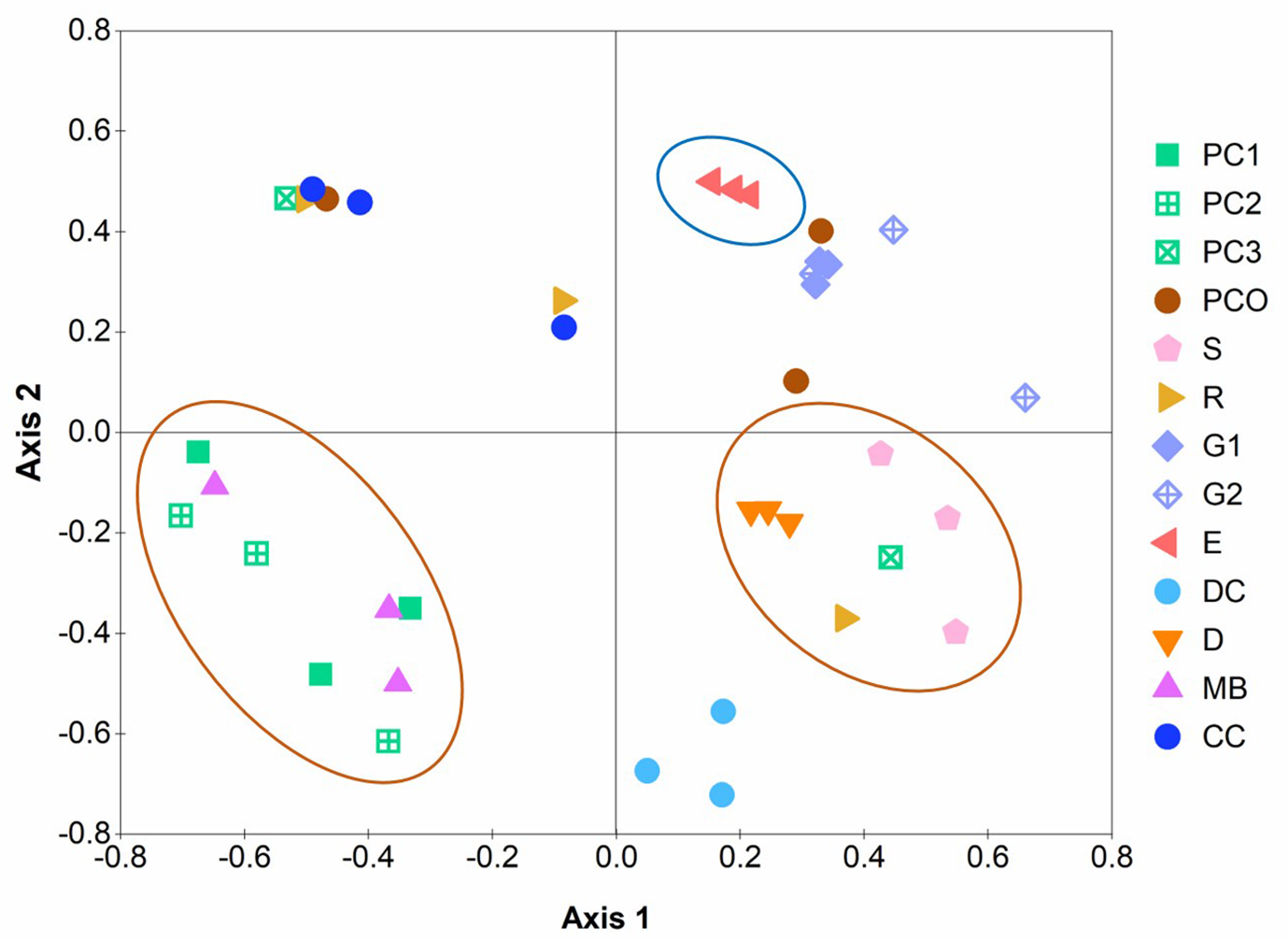


**Supplemental Figure 3.** qPCR for quantification of *nirS* and *alkB* against *16S rRNA* in E, D and MB.


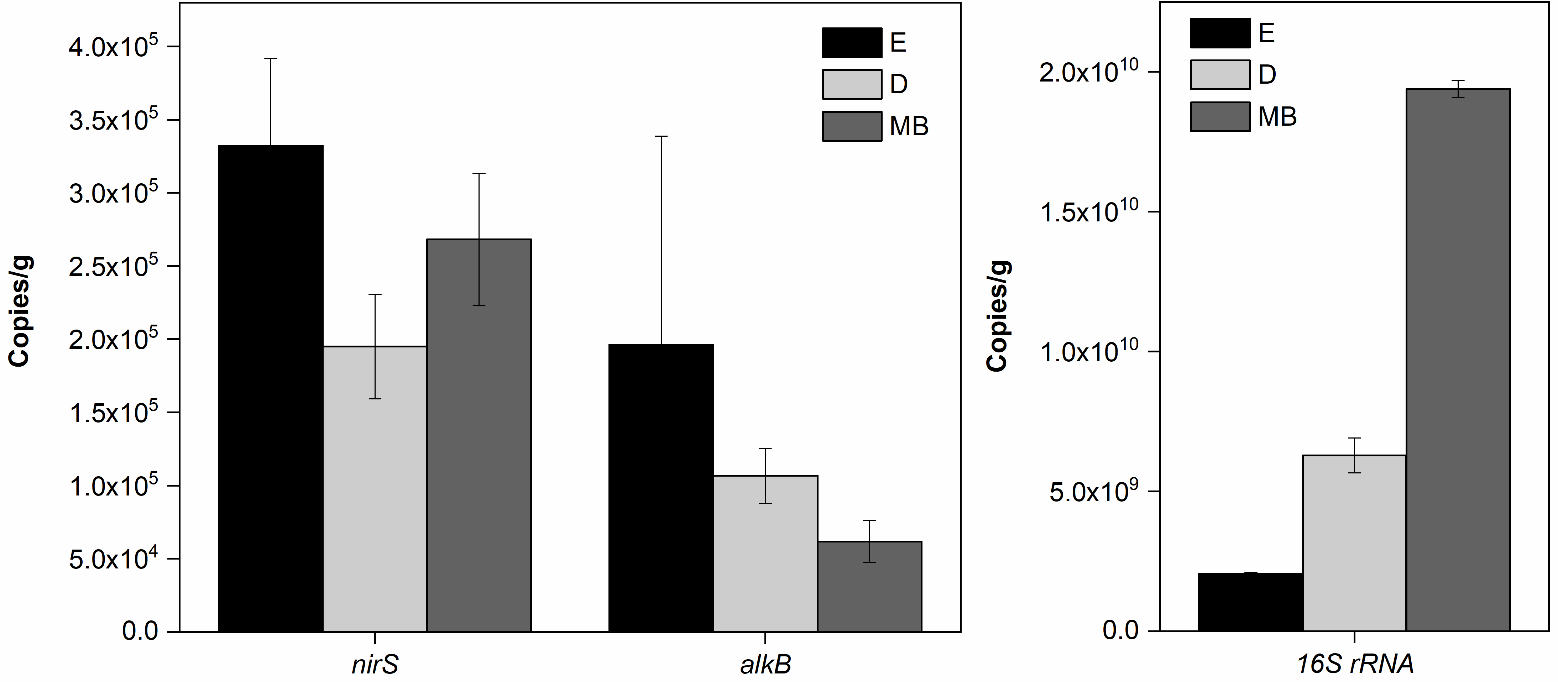


**Supplemental Table 1.** Average Good's coverage and alpha diversity indices from three independent replicates of bacterial taxonomic profiles by 16S rRNA gene sequences, at the (**a**) genus and (**b**) species levels.

**a**

| **Fecal manure** | **Raw read** | **Quality reads** | **OTUs** | **Good’s coverage** | **Chao** | **Shannon** |
| --- | --- | --- | --- | --- | --- | --- |
| PC1 | 379,447 | 247,429 | 141 | 0.9957 | 167.47 | 3.0624 |
| PC2 | 263,326 | 171,406 | 142 | 0.9948 | 217.64 | 2.8173 |
| PC3 | 321,526 | 209,245 | 110 | 0.9964 | 141.56 | 2.8481 |
| PCO | 220,694 | 125,493 | 107 | 0.9969 | 128.27 | 2.9622 |
| S | 233,745 | 170,280 | 87 | 0.9968 | 132.00 | 2.7005 |
| R | 169,150 | 158,649 | 133 | 0.9954 | 177.82 | 2.9170 |
| G1 | 85,006 | 53,119 | 92 | 0.9977 | 107.30 | 2.5213 |
| G 2 | 22,427 | 8,279 | 93 | 0.9974 | 113.84 | 2.4020 |
| E | 37,338 | 13,959 | 293 | 0.9877 | 407.99 | 3.3410 |
| DC | 126,839 | 97,494 | 112 | 0.9962 | 153.12 | 2.4019 |
| D | 189,084 | 110,329 | 142 | 0.9952 | 183.18 | 3.2547 |
| MB | 137,903 | 129,489 | 132 | 0.9956 | 173.44 | 2.8511 |
| CC | 165,464 | 155,851 | 141 | 0.9953 | 178.40 | 2.7594 |

**b**

| **Fecal manures** | **OTUs** | **Good’s coverage** | **Chao** | **Shannon** |
| --- | --- | --- | --- | --- |
| PC1 | 161 | 0.994462 | 211.5711 | 3.15285 |
| PC2 | 158 | 0.993832 | 224.9286 | 2.879344 |
| PC3 | 125 | 0.995343 | 178.9569 | 2.991143 |
| PCO | 133.6667 | 0.994714 | 194.367 | 3.140491 |
| S | 107.3333 | 0.99572 | 163.4613 | 2.787121 |
| R | 149.3333 | 0.995007 | 190.406 | 3.06889 |
| G1 | 111.3333 | 0.99635 | 148.4545 | 2.561228 |
| G2 | 107 | 0.99635 | 141.747 | 2.418816 |
| E | 316.6667 | 0.985484 | 471.1535 | 3.354084 |
| DC | 120.3333 | 0.996014 | 156.0769 | 2.46303 |
| D | 169.6667 | 0.993203 | 238.0567 | 3.319442 |
| MB | 154.3333 | 0.994672 | 191.3897 | 2.928702 |
| CC | 157.3333 | 0.994714 | 200.6552 | 2.916145 |
